# Distribution and habitat characterization of the recently introduced invasive mosquito *Aedes koreicus* [*Hulecoeteomyia koreica*]*,* a new potential vector and pest in north-eastern Italy

**DOI:** 10.1186/1756-3305-6-292

**Published:** 2013-10-10

**Authors:** Fabrizio Montarsi, Simone Martini, Marco Dal Pont, Nicola Delai, Nicola Ferro Milone, Matteo Mazzucato, Fabio Soppelsa, Luigi Cazzola, Stefania Cazzin, Silvia Ravagnan, Silvia Ciocchetta, Francesca Russo, Gioia Capelli

**Affiliations:** 1 Istituto Zooprofilattico Sperimentale delle Venezie, Viale dell’Università, Padua, Legnaro 10; 35020, Italy; 2 Entostudio snc, Via Buffa, Padua, Brugine 49; 35020, Italy; 3 ULSS 1, Public Health Department of Belluno Via Feltre, Belluno 57; 32100, Italy; 4 ULSS 2, Public Health Department of Feltre, Via Bagnols sur Ceze, Feltre, BL 3; 32032, Italy; 5 Veneto Region, Direzione Prevenzione, Servizio Promozione e Sviluppo Igiene e Sanità Pubblica, Dorsoduro, Venice 3493, Italy

**Keywords:** *Aedes koreicus*, Invasive mosquito species, Entomological surveillance, North-eastern Italy

## Abstract

**Background:**

The container breeding species belonging to the genus *Aedes* (Meigen) are frequently recorded out of their place of origin. Invasive *Aedes* species are proven or potential vectors of important Arboviruses and their establishment in new areas pose a threat for human and animal health. A new species of exotic mosquito was recorded in 2011 in north-eastern Italy: *Aedes* (Finlaya) *koreicus* [*Hulecoeteomyia koreica*]. The aim of this study was to characterize the biology, the environment and the current distribution of this mosquito in north-eastern Italy. Morphological details useful to discriminate this species from other invasive *Aedes* mosquitoes are also given (see Additional files).

**Methods:**

All possible breeding sites for larval development were monitored. In addition, ovitraps and traps for adults were used to collect eggs and adults. The mosquitoes (larvae and adults) were identified morphologically and molecularly. Environmental data and climatic variables during the period of mosquito activity (from April to October) were considered.

**Results:**

*Aedes koreicus* was found in 37 municipalities (39.4%) and was detected in 40.2% of places and in 37.3% of larval habitats monitored, in a range of altitude from 173 to 1250 m.a.s.l.. Garden centres were the most common locations (66.7%), followed by streets/squares (57.1%), private gardens (46.4%) and cemeteries (21.1%) (p < 0.01). The main larval habitats were catch basins (48.5%) and artificial water containers (41.8%). As for *Aedes albopictus* [*Stegomyia albopicta*], ovitraps were attractive for adult females resulting in the higher rate of positivity (15/21; 71.4%) among breeding sites. The period of *Ae. koreicus* activity ranged from March 29 to October 29.

**Conclusion:**

The species is clearly established in the area and is now overlapping with other vectors such as *Ae. albopictus* and colonizing areas over 800 m.a.s.l, not yet or sporadically reached by the tiger mosquito. The data collected are essential to assess the risk of colonization of other parts of Italy and Europe, as well as the risk of spreading of pathogens transmitted. These findings stress the importance of implementing entomological surveillance for early detection of invasive species, which is necessary for eradication or limitation of its further spread.

## Background

The global trade, the increase of travel and the improvement of connection among people, along with climatic and environmental changes, increase the risk of the introduction and adaptation of arthropod vectors to new environments [1]. In particular, the container breeding species belonging to the genus *Aedes* (Meigen) are frequently recorded out of their place of origin, mainly due to their diapausing eggs that survive to desiccation during the passive transport [2]. Invasive *Aedes* species are proven or potential vectors of important Arboviruses [3,4] and their establishment in new areas pose a threat for human and animal health.

The tribe Aedini has recently been reclassified by authoritative scientists [5-8]; however, in this article, the traditional names have been used in association with new names in parentheses.

Italy is characterized by a temperate climate and an environment that offers many favourable habitats to different species of mosquitoes; therefore invasive *Aedes* mosquitoes may have the opportunity to establish after their introduction [9]. The Asian tiger mosquito, *Aedes albopictus* (Skuse) [*Stegomyia albopicta*] represents the best example of colonization and spreading of an *Aedes* species in Europe [10]. Since the discovery of the first established population of tiger mosquito in 1991 in north-eastern Italy [11], many local surveillance and control programs were carried out. During the routine monitoring for tiger mosquito in the Veneto Region, in 2011 several *Aedes* larvae were collected from a catch basin in a small village of the province of Belluno, northern Italy, an area still considered free from *Ae. albopictus*. Larvae and adults reared in laboratory were then morphologically and molecularly identified as *Aedes* (*Finlaya*) *koreicus* [*Hulecoeteomyia koreica*] [12,13], a species native of south-east Asia.

After this record, a more intensive surveillance was gradually extended from positive sites in surrounding areas to verify the distribution of this species, to attempt to trace back the possible route of entry and to collect biological data on its life cycle. In this work we report the results of two years of surveillance and the implication for the monitoring and control of a new species, which overlapped with another similar invasive mosquito, *Ae. albopictus*. A quick guide for the identification of the species, reporting the main morphological features of the Italian population of *Ae. koreicus,* is included as Additional files, to help personnel involved in the entomological monitoring with a prompt recognition of this species.

## Methods

### Study area

**Figure 1:**
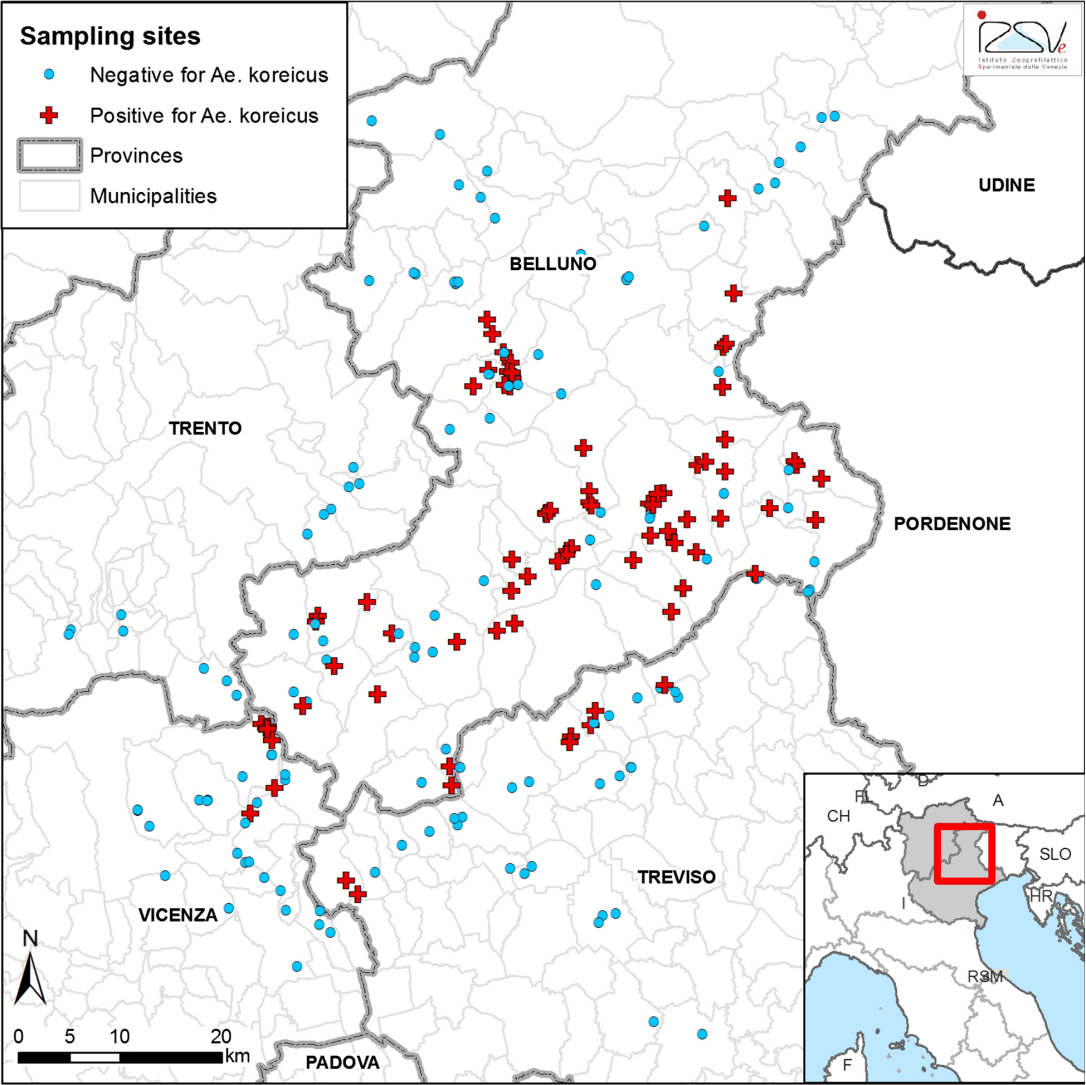
**Map of the monitored area and sampling sites in north-eastern Italy, 2011–2012.** Legend: negative (blue dots) and positive sites (red cross) for the presence of *Aedes koreicus.*

The monitoring started in 2011 from the valley (Valbelluna, Province of Belluno, northern Italy) where the first mosquitoes were found and was extended in the whole Province of Belluno and in the neighbouring Province of Vicenza, Treviso and Trento in 2012 (Figure 1).

The valley is surrounded by Bellunesi Prealps and Dolomites, extending from north-east to south-west for 50 kilometres with an average elevation of 527.7 m.a.s.l.. The area has a sub-continental climate, characterized by temperate climate, with cold and often snowy winters and mild warm summers. The annual rainfall is above 1,000 mm. The Province of Belluno (3,600 km^2^) is an area of Veneto Region neighbouring Friuli Venezia Giulia and Trentino Alto Adige regions (Italy) and Austria. The population density in the province is one of the lowest of Italy (57.7 inhabitants/km^2^). The villages are quite small and only two have more than 10,000 inhabitants (Feltre and Belluno). The rest of the area monitored (Province of Vicenza, Treviso and Trento) is characterized by climatic and environmental conditions similar to Valbelluna valley.

### Environmental and climatic data

Environmental data were obtained from Land Use Map (CORINE Land Cover 2006). Vegetation is dominated by Beech (*Fagus sylvatica*), Downy Oak (*Quercus pubescens*) and European Hophornbeam (*Ostrya carpinifolia*). Coniferous and pine forests are present on the mountains. Water bodies and lakes are abundant at the lowest elevation. The area is mainly covered by forest or natural vegetation (75.0%) and little used for agricultural activities (20.2%); the water bodies represent 0.5% of the area and only the 4.3% of the area is urbanized. The human settlings are small villages composed by country houses with private gardens and public parks, which offer many breeding possibilities to container breeding mosquitoes of the *Aedes* genus.

Climatic data of the area monitored were obtained from the Meteorological Regional Centre [14] from 4 meteorological stations (Belluno, Feltre, Sospirolo, S. Giustina). Monthly means of minimum, mean and maximum daily temperatures and accumulated monthly precipitation were calculated. The climatic variables of the study area were compared with the data of South Korea [15] where the mosquito is endemic. The broadest possible long-term period was considered to characterize the climate of the areas: from 1994 to 2011 for the study area and from 1981 to 2010 for South Korea.

### Mosquito sampling

All possible breeding sites, such as catch basins, man-made containers, buckets, basin of fountains, tires, vases/flowerpots and natural mosquito larval habitats (tree holes, water in plants, puddles) were checked. The places visited in the villages included private and public places, i.e. gardens, garden centres and florists, tire markets, cemeteries, farms and houses.

*Aedes* eggs were collected with standard ovitraps (black vases filled with 300 ml of water, 8 cm upper diameter) with sticks of masonite™ as support for oviposition.

Larvae collections were made using a standard larval dipper (500 ml, 10 cm diameter). The larvae collected were placed in 50 ml plastic tubes and transported alive to the laboratory in a cooler. The adults were collected using BG-Sentinel™ traps baited both by CO_2_ and lure™, testing also the ability of the traps to catch this species. During larval collection, adults trying to land on the personnel were also collected. Adults and larvae obtained from the hatching of eggs were examined.

The end of *Ae. koreicus* activity was defined when there were no more adults, larvae or positive ovitraps.

### Mosquito identification

The larvae collected (either hatched from ovitraps or from direct collections) were stored in 70% ethanol for further identification. A small number of larvae were reared in the laboratory to obtain adults. The 4th-instar larvae were clarified in saturated solution of alcohol-phenol for 4-5 h and then transferred in phenol-balsam (adding few drops of alcohol-phenol in Canada balsam) used as mounting medium [16]. The same procedure was applied to examine genitalia of males. The specimens were morphologically identified [12,17-22].

Six females and six males were examined accurately; the legs were clarified, mounted on slides and length of white band measured. Twelve larvae were also examined. The specimens described were collected as larvae during the summer from two villages where only *Ae. koreicus* was present or obtained from eggs hatched in laboratory.

Particular attention was paid to the morphological features useful to make a correct and rapid identification of larvae and adults of *Ae. koreicus* (see Additional file 1**)**. *Aedes koreicus* was compared with the most similar species *Aedes (Finlaya) japonicus* (Theobald) [*Hulecoeteomyia j. japonica*] [17,18,23] and *Ae. albopictus.* The comparative study reports only the features that differ among the three species (see Additional file 2). Drawings of morphology and terminology of the characters described are provided (see Additional files 3 and 4). Finally, photographs of *Ae. koreicus* are compared to *Ae. albopictus* to draw attention to their differences (see Additional files 5, 6, 7 and 8**)**.

In case of doubtful morphological identification or findings in new areas, a molecular confirmation was carried out using a PCR targeting the nicotinamide adenine dinucleotide dehydrogenase (NADH) subunit 4 (ND4) gene and using the primers and protocol suggested by [13,24].

### Statistical analysis

The chi-square test was used to test significance of the differences of *Ae. koreicus* prevalence (only place/breeding sites monitored more than 10 times) according to the place of collection, larval breeding sites, altitude range. Prevalence distribution of *Ae. koreicus* was also compared with *Ae. albopictus* according to altitude.

## Results

### Distribution, habitat and period of activity

The area was monitored from the discovery of the species, on May 24 through September 2011 and from March through October 2012. The altitude of the sampling places ranged from 14 to 1645 m.a.s.l..

A total of 94 municipalities were monitored: 53 in the Province of Belluno (76.8%) and 41 in other villages in the Province of Vicenza, Treviso and Trento (Table 1). Overall, 290 collections were made (241 larval and 15 adult collections and 34 ovitraps) and mosquitoes belonging to different species were found in 213 cases (73.4%) of all collections; only in four villages no mosquitoes were collected. Overall, 229 different potential aquatic habitats were monitored, i.e. 86 pools of vases (each pool constituted of 30–40 vases) in cemeteries, 67 water containers in garden centres, private and public gardens, 33 catch basins in streets or squares.

**Table 1:** **Municipalities monitored and positive for *Aedes koreicus *in 2011–2012 in north-eastern Italy**

**Year and provinces**	**Municipalities monitored/total (%)**	**Municip. positive/monitored for *Aedes koreicus *(%)**
*2011*		
Belluno	23/69 (33.3)	17/23 (73.9)
Treviso	2/95 (2.1)	0/2 (0.0)
*2012*		
Belluno	45/69 (65.2)	23/45 (51.1)
Treviso	18/95 (18.9)	4/18 (28.6)
Vicenza	13/121 (10.7)	3/13 (25.0)
Trento	8/217 (3.7)	0/8 (0.0)
**Total**	**94/502 (18.7)**	**37/94 (39.4)**

*Ae. koreicus* was found in 37 municipalities out of 94 monitored (39.4%) in a range of altitude between 173 (Valstagna, Province of Vicenza, Lat 45°53′10.72″N; Long 11°41′3.73″E) and 1,250 m.a.s.l. (Pedavena, Province of Belluno: Lat 46° 04′ 12″; Long 11° 50′ 31″). *Ae. koreicus* was detected in 40.2% of the locations and in 38.0% of the larval habitats checked. Garden centres were the most common locations (66.7%), followed by streets/squares (57.1%), private gardens (46.4%) and cemeteries (21.1%) (Table 2).

**Table 2:** **Results of the monitoring for *Aedes koreicus *in 2011–2012 in north-eastern Italy**

**Locations**	Positive/monitored for *Aedes koreicus *(%)*	**Breeding sites**	Positive/monitored for *Ae. koreicus *(%)*
Cemeteries	19/90 (21.1)^ABC^	Vases	16/86 (18.6%)^ABC^
Garden centres	12/18 (66.7)^A^	Water containers	28/67 (41.8%)^Ad^
Private gardens	13/28 (46.4)^B^	Catch basins	16/33 (48.5%)^B^
Public parks	2/6 (33.3)	Puddles	3/4 (75%)
Tire markets	4/8 (50.0)	Tires	4/9 (44.4%)
Streets/squares	20/35 (57.1)^C^	Basin of fountains	5/7 (71.4%)
Depots	4/5 (80.0)	Ovitraps	15/21 (71.4%)^Cd^
Buildings	5/7 (71.4)	Dunghill	0/1 (0.0%)
Farms	1/4 (25.0)	Tree holes	0/1 (0.0%)
Forests	2/3 (66.7)		
**Total**	**82/204 (40.2%)**	**Total**	**87/229 (38.0%)**

Positive and surveyed locations for the presence of larval breeding sites and kind of larval breeding sites (in parentheses percentage of positive).

*Significant differences are marked with the same letter (uppercase = p < 0.01; lowercase = p < 0.05)

The main larval habitats found positive for *Ae. koreicus* larvae were catch basins (48.5%) and artificial water containers (41.8%). As for *Ae. albopictus*, ovitraps were attractive for adult females resulting in the higher rate of positivity (15/21; 71.4%) among breeding sites (Table 2). Larvae were also observed in a turtle tank located inside a house.

Six other mosquito species were identified in 152 collections (59.4%) (excluding ovitraps) during the survey: *Culex pipiens* (L.) (42%), *Cx. hortensis* (Ficalbi) (2.3%), *Aedes geniculatus* (Olivier) [*Dahliana geniculata*] (0.8%), *Culiseta longiareolata* (Macquart) (0.8%) and *Anopheles maculipennis s.l.* (Meigen) (0.4%). *Aedes albopictus* was found in 57 (23.8%) out of 225 breeding sites monitored (including ovitraps), even in places not previously colonized.

**Figure 2:**
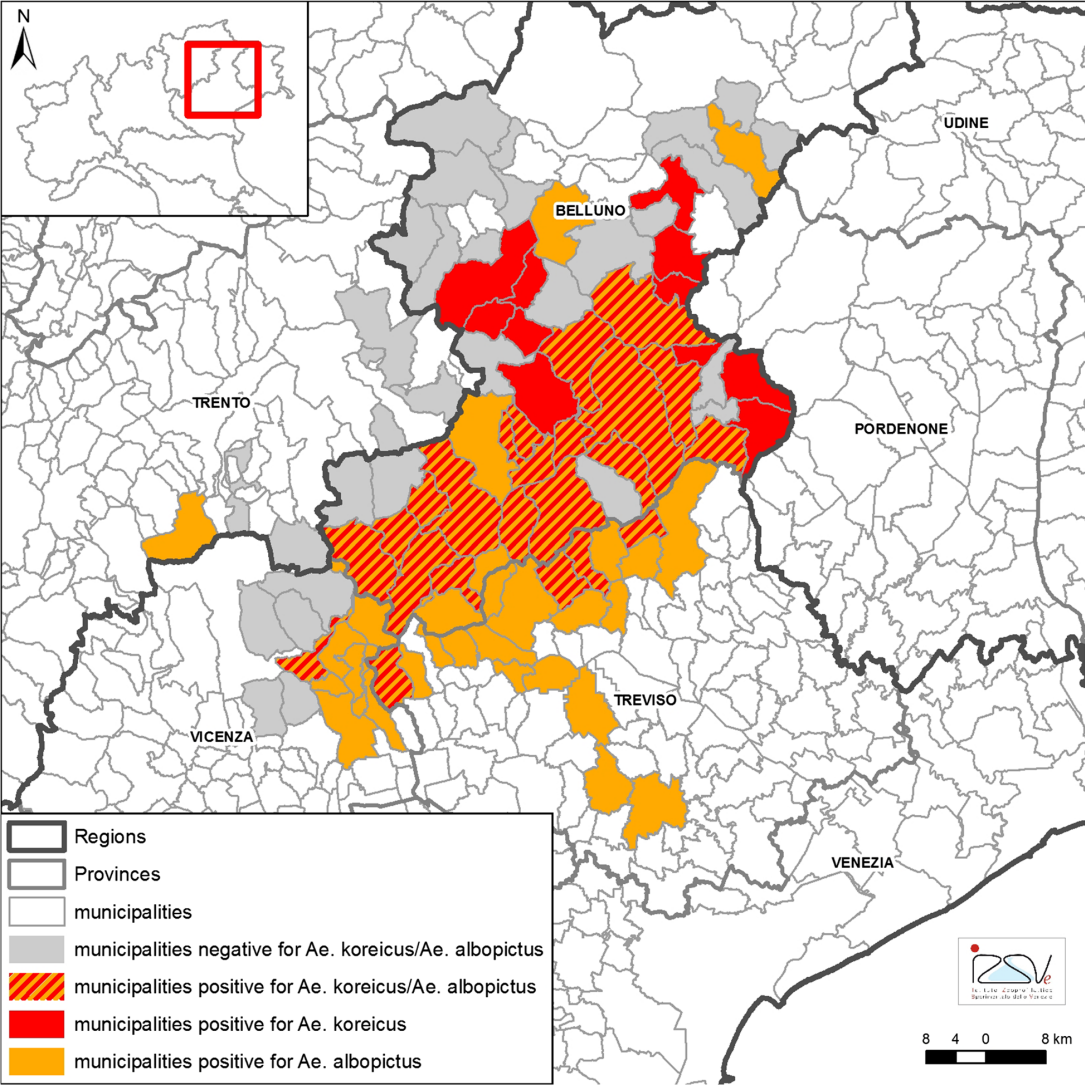
**Map of monitored municipalities in north-eastern Italy, 2011–2012.** Legend: municipalities positive for the presence of *Aedes koreicus*, *Aedes albopictus* and their overlapping areas.

**Figure 3:**
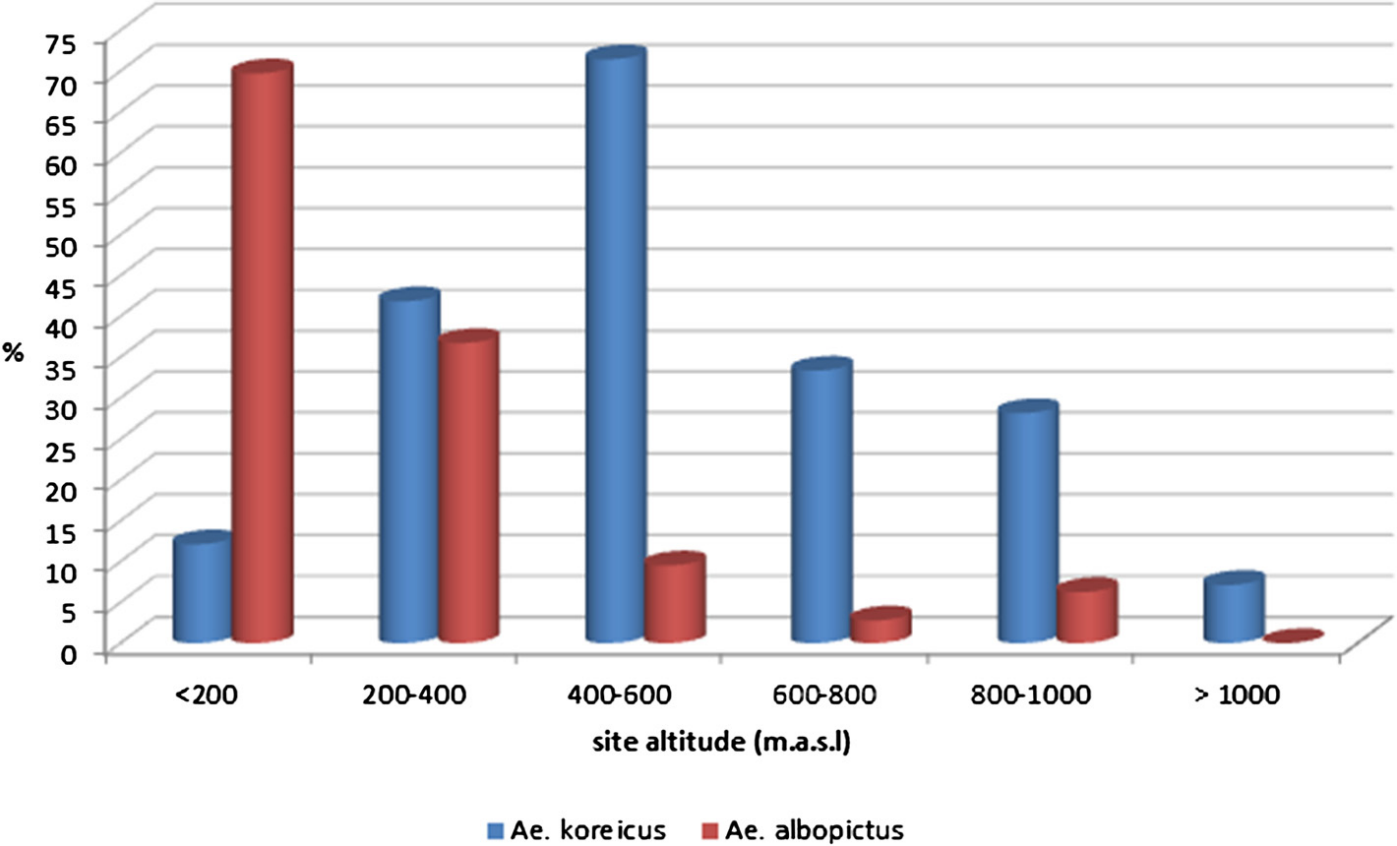
**Distribution of *Aedes koreicus *and *Aedes albopictus *according to altitude in north-eastern Italy, 2011–2012.**

Out of 113 collections positive for *Ae. koreicus,* larvae were identified as the only species in 80 cases (71%) and were associated with *Cx. pipiens* 17 times (including 1 ovitrap, 15%), *Ae. albopictus* 13 times (including 4 ovitraps, 11.5%), *Cx. hortensis* six times (5.3%) and *Ae. geniculatus* in one site. In 21 municipalities (22.3%) both *Ae. koreicus* and *Ae. albopictus* were present (Figure 2). *Aedes koreicus* was more common in locations between 400–600 m.a.s.l. (71.4%) whereas *Ae. albopictus* was more common in places under 200 m.a.s.l. (61.9%) (p < 0.01) (Figure 3).

*Ae. koreicus* was also well represented at an altitude between 800–1000 m.a.s.l. (28.1%) and was found in two sites above 1000 m.a.s.l., where *Ae. albopictus* was absent.

In 2011 the beginning of the *Ae. koreicus* activity remained unknown as the survey started after the first identification at the end of May; the last adults were collected on September 13, while ovitraps remained positive until October 13. In 2012 the first larvae were caught on March 29 and ovitraps were positive on May 5. The adults were active until the end of September and living larvae were observed until October 29. The period of activity of *Ae. albopictus* was July 7-September 8 in 2011 and May 23-September 28 in 2012.

**Figure 4:**
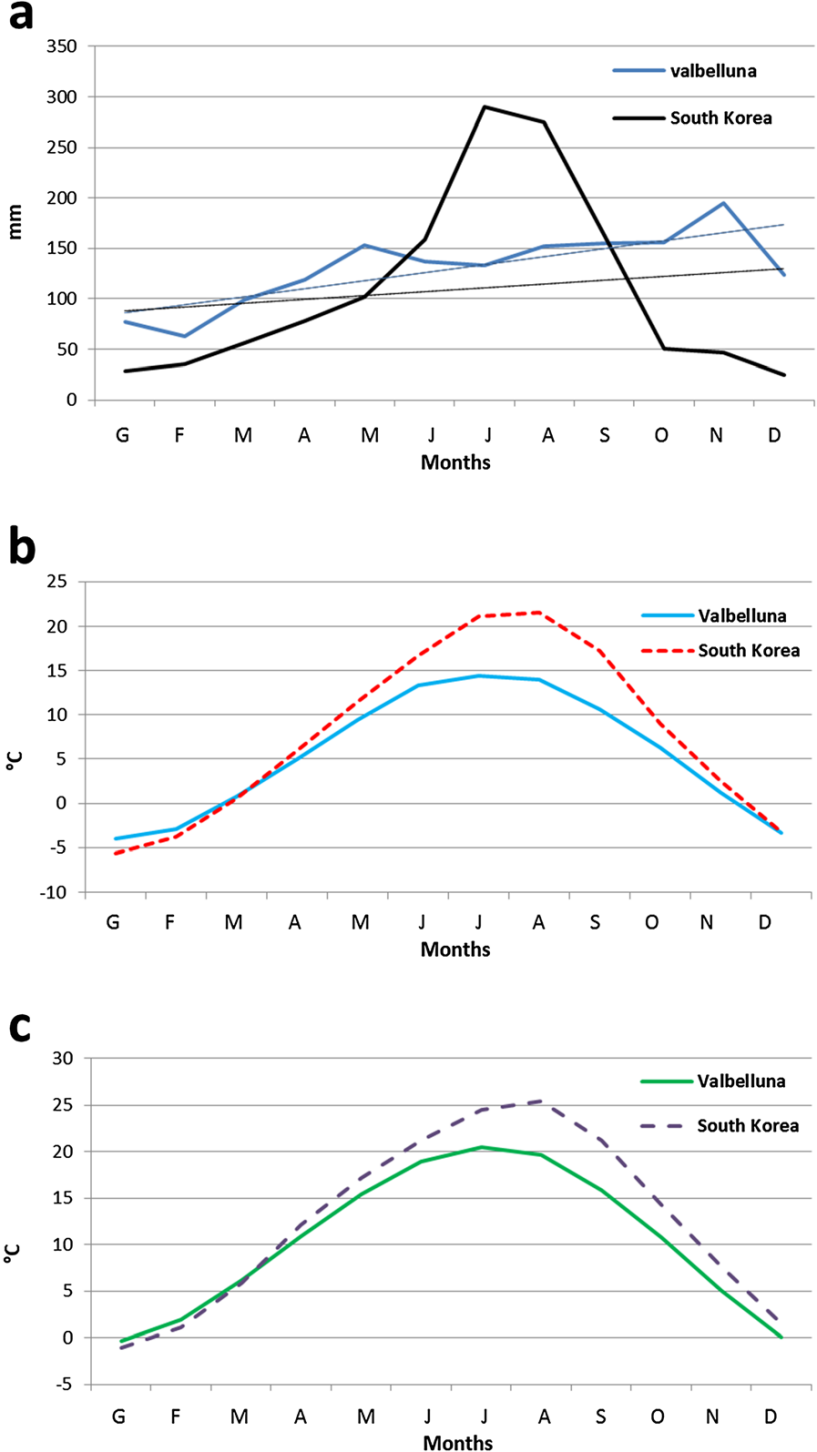
**Climatic variables of the study area and native area of *Aedes koreicus *(South Korea).** Legend: Monthly means of precipitation (curves: monthly precipitation; lines: trend lines) **(a)**, mean temperature **(b)** and minimum temperature **(c)** of the Valbelluna (study area) and South Korea (native area).

The mean temperature at the beginning and at the end of the period of *Ae. koreicus* activity (April-October) was 10.9°C and 10.8°C and the mean minimum temperature in the same period was 4.9°C and 6.2°C. The temperatures in South Korea showed the same trend (mean temperature range 12.2-14.3°C; minimum temperature range 6.0-9.0°C) while the annual rainfall in Valbelluna is higher than in South Korea (1,500 *vs.* 1,000 mm) (Figure 4).

In August 2012, *Ae. koreicus* adults were caught by one out of three CO_2_ baited BG-Sentinel traps but not by the two traps with only odorous attractant. Adults were also captured inside a house up to the fourth floor and in 2 of 3 forests where no buildings were present.

### Molecular identification

All doubtful samples were identified by PCR and yielded the expected band of 283 bp. The sequences revealed 99-100% identity with the Belgium isolate (GenBank:JF430392) and with the South Korea isolates (GenBank:GU229925-27). The sequence of the first finding of *Ae. koreicus* in Italy (May 2011) and two representative sequences of our samples were submitted to GenBank database (GenBank: KC551970, KC551968 and KC551969, respectively).

## Discussion

The results of our survey clearly show that *Ae. koreicus* is well established in an area of 2600 km^2^ of north-eastern Italy and confirm the invasive potential of this mosquito [24].

The finding of *Ae. koreicus* in Italy represents the second incursion in Europe, after a previous report in Belgium [25]. Outside Europe, *Ae. koreicus* is common in Korea and China and reported in Japan [23,26] and in the Asian part of Russia [27]. However, the current distribution of the species in Asia is unclear due to the lack of recent studies; furthermore *Ae. koreicus* was considered being a variety of *Ae. japonicus* and was often confused in the past with similar species [17]. Only recently its distribution in Korea has been defined [21] and the taxonomic position clarified using molecular tools [24].

Alien species need some time to adapt to their new environment and seem to follow a three-step process to settle and expand, i.e. arrival, establishment (population growth and unlikely extinction) and spread (expansion into new areas) [28,29]. The current scenario of *Ae. koreicus* in Italy seems to represent the third phase, characterized by occasional long-distance dispersal and formation of isolated new colonies [28,30]. The probability of establishment and spread of invasive species depends initially on suitable climatic conditions [31], on the availability of aquatic habitats (larval breeding sites) [32-34] and on the presence of suitable hosts. The knowledge of these features is of particular importance to predict their future spread.

Little information on the biology and the behaviour of *Ae. koreicus* in an area out of the native area is available [25,35]. In the native area it colonizes a wide typology of natural and artificial containers and seems more abundant in urban areas than *Ae. japonicus*[18]. It can tolerate the cold winter temperature and the first larvae are found early in the spring [17]. The area colonized in Italy displays air temperatures and freezing days during winter similar to the native South Korea. As opposed to *Ae. albopictus*, the cold season is not a limiting factor to the establishment and spread of *Ae. koreicus*, thus extending the risk of invasion all over central Europe.

Based on our observations, *Ae. koreicus* confirms also to be well adapted to urban settlements. The main sites colonized are garden centres, urban areas (streets, squares, parking lots) and private gardens (flower and kitchen gardens, sets of flowers on the balcony) where it breeds in a variety of artificial man-made containers. Surprisingly larvae were also found in fountains located into the forest, far from human settlings, indicating that the species is able to complete its life cycle feeding on animals other than humans.

Another important role in the process of settling and spreading for the new species, apart from the climate and the environment, is played by the competition with the pre-existing species [32,33,36-38], which takes place mainly during the aquatic larval phase [33]. *Aedes koreicus* has arrived in an area in part occupied by indigenous mosquito species mainly belonging to the species *Cx. pipiens*, *Cx. hortensis*, *Ae. geniculatus* and *Ae. albopictus*. The most likely competitor of *Ae. koreicus* is surely *Ae. albopictus*, which uses similar larval habitats for the development and is considered superior to other mosquitoes in resource competition [32,36,37,39-43]. According to our observations, larval coexistence between *Ae. koreicus* and *Ae. albopictus* is possible but not common. In shared breeding sites, the number of *Ae. albopictus* larvae was higher than *Ae. koreicus* during the fall and toward south. The latter species was dominant in cohabitation just in a single case at high altitude. The unusual persistence of *Ae. koreicus* in artificial containers in the presence of *Ae. albopictus* may be facilitated by differences in seasonality and colonization to higher altitude areas. Indeed, *Ae. koreicus* adults hatch in the spring much earlier than *Ae. albopictus* (March and end of May respectively) leading to larval population of bigger size, as observed for *Ae. japonicus* and *Ae. albopictus*[42].

However, in the majority of the cases, *Ae. koreicus* was the only species found, demonstrating it has preferentially occupied empty niches [44].

The standard ovitraps used for tiger mosquito monitoring and the CO_2_ baited BG-sentinel trap were shown to be suitable for *Ae. koreicus* collection. Accordingly, the current surveillance system is now complicated by the presence of two similar species in the same environment, thus requiring well trained personnel for identification.

It was not possible to clearly demonstrate the time of arrival and the route of entry of the mosquito in this part of Italy. Based on the previous monitoring for *Ae. albopictus* in the same area [45,46]. *Ae. koreicus* was apparently absent or undetectable before 2010. However, comparing the size of this area with the area colonized by *Ae. japonicus* in central Europe (approx. 2,600 *vs.* 1,400 km^2^), where *Ae. japonicus* has been present from at least since 2008 [47], it can be assumed that this species was likely introduced 3–4 years ago.

All the specimens collected (larvae and adults) showed the same morphological and molecular characteristics of the Belgian population and of the Jeju-do Island of Korea population [25], suggesting a possible introduction from one of these locations. The way of introduction was likely through eggs within small containers, tires or plants, as happened in the past for *Ae. albopictus*[2,29,48,49].

It’s interesting to note that this area of north-eastern Italy and in particular the Veneto Region has experienced several invasive mosquito introduction and spread in the past 20 years; in particular, *Ae. albopictus* in 1990, which spread all over the country in a few years [9,11], *Ae. atropalpus* [*Georgecraigius atropalpus*] in 1996 [50], detected in the Province of Treviso and eradicated, and now *Ae. koreicus*. This is likely a consequence of the intensive trade of goods in one of the most developed industrial and commercial area of Italy. However, the intensive mosquito monitoring that followed recent vector-borne outbreaks in north Italy [51-53] could have enhanced our probability to detect invasive mosquitoes. Trade of goods and road vehicles movement are also likely the route of local dispersal, as happened with *Ae. albopictus*[9].

Other aspects of the biology of *Ae. koreicus* need urgently to be investigated in the next future, such as the feeding behaviour and the vector competence. The people resident in the areas where *Ae. koreicus* is the exclusive diurnal biting species complained due to mosquito bites (behaviour confirmed by the authors). Except humans, the host preference spectrum in this area remains unknown.

Little information on *Ae. koreicus* vector competence is available; while experimental transmission of Japanese encephalitis virus (JEV) has been proven [54-56], the virus has not been isolated from wild-caught mosquitoes in Korea [57]. There is also laboratory evidence of its involvement in the parasitic cycle of the dog heartworm *Dirofilaria immitis*[58], which is endemic in north-eastern Italy and mainly transmitted by *Culex* and *Aedes* mosquitoes [59,60].

Some arboviruses, such West Nile and Usutu viruses, are endemic in Veneto region [51,52] and others, such as Chikungunya and Dengue viruses, are regularly introduced by infected humans every year [61]. If *Ae. koreicus* will be demonstrated to be competent for some of these viruses, the risk of transmission will be extended in this new colonized area, especially at high altitudes, i.e. in places previously regarded at a negligible risk of animal and human outbreaks.

## Conclusions

The findings of this survey defined horizontal and vertical geographical limits of the area colonized by *Ae. koreicus* in northern Italy and reported some biological characteristics of the species, such as period and temperatures of activity and breeding sites preference. These data are important to model the risk of spreading in other part of Italy or Europe and to plan the future control of this invasive species, considering that the eradication of an establish species is unlikely.

Other research is urgently needed, especially to study the host preference spectrum and the vector competence of the species, to define the risk of infection transmission.

This finding, once again, stresses the importance of implementing an integrated entomological surveillance system for early detection of invasive species, which is necessary for eradication or limitation of its further spread.

## Competing interests

The authors declare that they have no competing interests.

## Authors’ contributions

FM, SM and GC conceived the study. FM, SM, MDP, ND, NFM, SCi carried out the samplings. SR and SCa carried out the molecular study. GC performed the statistical analysis. MM made the maps. FS, LC and FR supported this study. FM took the pictures and drew the drawings in Additional files 3 and 4. FM carried out the morphological identification and wrote the first draft of the manuscript. GC reviewed the manuscript. All authors read and approved the final manuscript.

## Supplementary Material

Additional file 1 **Morphological features of *Aedes koreicus. ***Description of the main characteristics of adults and larvae of *Aedes koreicus*, (Italian or Belgian specimens). Click here for file

Additional file 2: Table S3 A quick guide for the morphological identification of larvae and adults of *Aedes koreicus*, compared to *Aedes albopictus* and *Aedes japonicus*. Table showing the main characteristics of *Aedes koreicus* (Italian or Belgian specimens), *Aedes albopictus* and *Aedes japonicus (*according to [18]). Click here for file

Additional file 3 **Drawings of the main characteristics considered for the identification of female culicinae mosquito.** a - general aspect of a female mosquito; b – dorsal and lateral view of thorax. The arrows point only the characteristics useful for the identification of *Aedes koreicus* and *Aedes albopictus.* Click here for file

Additional file 4 **Drawings of main characteristics considered for the identification of larvae and male of *Aedes *species.** c – hypopygium of *Aedes*; d – head of culicinae larvae; e – lateral view of the distal part of the larval abdomen of *Aedes/Ochlerotatus* species. The arrows point only the characteristics useful for the identification of *Aedes koreicus* and *Aedes albopictus.* Click here for file

Additional file 5 **Comparison between larval features of *Aedes albopictus *and *Aedes koreicus. ***Difference between *Aedes albopictus* (a) and *Aedes koreicus* (b); **Figure S5**, antennae and antennal setae 1A; **Figure S6**, comb scales; **Figure S7**, pecten. Click here for file

Additional file 6 **Comparison between male features of *Aedes albopictus *and *Aedes koreicus. ***Difference between *Aedes albopictus ***(a)** and *Aedes koreicus* (b); **Figure S8**, palps and proboscids; **Figure S9**, hypopygium. Click here for file

Additional file 7 **Comparison between female features of *Aedes albopictus *and *Aedes koreicus. ***Difference between *Aedes albopictus* (a) and *Aedes koreicus* (b); **Figure S10**, palps and interorbicular space; **Figure S11**, ornamentation on scutum; **Figure S12**, abdomen. Click here for file

Additional file 8 **Comparison between legs of *Aedes albopictus *and *Aedes koreicus. ***Difference between *Aedes albopictus* (a) and *Aedes koreicus* (b); **Figure S13**, 1-2-3 fore, mid and hind legs respectively. Click here for file
