# Supplementary material for: Distribution and habitat characterization of the recently introduced invasive mosquito Aedes koreicus [Hulecoeteomyia koreica], a new potential vector and pest in north-eastern Italy
Source: Parasit Vectors. 2013 Oct 10;6:292. doi: 10.1186/1756-3305-6-292 (PMC3852218; doi:10.1186/1756-3305-6-292)
Supplement: Additional file 1 — Morphological features of Aedes koreicus. Description of the main characteristics of adults and larvae of Aedes koreicus, (Italian or Belgian specimens). [file 1756-3305-6-292-S1.doc]

**Morphological features**

The main differences on morphological characters among adults and larvae of *Ae. koreicus*, *Ae. japonicus* and *Ae. albopictus* are reported in table 3 (see **additional file 2)**. According to [18] small morphological variations between *Ae. koreicus* specimens from Korean Peninsula and Jeju-do island exist. Recently, a detailed description of *Ae. koreicus* detected in Belgium was reported [25], indicating that the specimens found in Belgium show the pattern as the Jeju-do individuals [57]. The specimens examined in this study show the same pattern as the Belgian individuals.

The general aspect of adults of the three species is similar: a dark mosquito with white strips on body and legs. In detail, *Ae. albopictus* presents white strips very brilliant while the other species have whitish strips. The feature more different is the ornamentation of the scutum: a single white median line in *Ae. albopictus* versus a distinctive pattern of pale scales arranged in complex painting. The following description is limited to some features of *Ae. koreicus* collected.

Larvae: (see **additional file 5; Figures 5, 6 and 7**) the head is orbicular not rugose. The inner and outer clypeal setae with 3 or more branches. Antennae covered with spicules; the antennal setae (1-A) is 2-3 forked and proximal to middle of the antenna (**Figure 5**). Thorax and abdomen without pilosity or stellate hairs. The comb scales are typical, about 45-60 in number, broadened apically and with lateral fringes (**Figure 6**). The pecten on siphon has 22-28 teeth evenly spaced each of which has 4-6 denticles; no detached simple pecten teeth were found beyond the base of siphonal tuft (**Figure 7**). The saddle shows a typical form with complex apical spines. The ventral bush has 1-2 tufts arising from the barred area.

Male: (see **additional file 6; Figures 8 and 9**) the palps are all dark longer than proboscis (**Figure 8**); the rest of ornamentation shows the same pattern as female. Genitalia have basistyle without distinct basal lobe, gonostylus shorter than basistyle with apical spine; clapsette stem with 3 distinct apical hairs; tergum IX lobes with about 7 hairs (**Figure 9**).

Female: (see **additional file 7; Figures 10, 11 and 12**) the proboscis and palps are black and the vertex has pale narrow scales extended along the eyes margin (**Figure 10**). The first antennal joint (pedicel) is dark with few distal pale scales. The scutum has a typical ornamentation (**Figure 11**). The subspiracular area bears a patch of pale scales. According to other authors [18,25] the abdomen shows a high degree of variation, but the pattern observed most frequently is the following: tergum I and VIII with a basomedian pale band, terga II-VII with basolateral and basomedian pale areas (**Figure 12**).

The legs ornamentation is the character more different among the three species compared. *Ae. albopictus* present white basal band broader than other species. In particular, the hind tarsum is the most useful feature to distinguish these mosquitoes; *Ae. koreicus* and *Ae. albopictus* have white basal band on tarsomere 1-4 that lacks in *Ae. japonicus* on tarsomere 4 (entirely dark). *Ae. albopictus* is the only one with the tarsomere 5 entirely white; it is black in *Ae. japonicus* and pale basal banded in *Ae. koreicus* (see **additional file 8; Figure 13**).
