# Supplementary material for: Distribution and habitat characterization of the recently introduced invasive mosquito Aedes koreicus [Hulecoeteomyia koreica], a new potential vector and pest in north-eastern Italy
Source: Parasit Vectors. 2013 Oct 10;6:292. doi: 10.1186/1756-3305-6-292 (PMC3852218; doi:10.1186/1756-3305-6-292)
Supplement: Additional file 2: Table S3 — A quick guide for the morphological identification of larvae and adults of Aedes koreicus, compared to Aedes albopictus and Aedes japonicus. Table showing the main characteristics of Aedes koreicus (Italian or Belgian specimens), Aedes albopictus and Aedes japonicus (according to [18]). [file 1756-3305-6-292-S2.docx]

| **Characteristics of adults** | ***Aedes albopictus*** | ***Aedes koreicus*** | ***Aedes japonicus*** |
| --- | --- | --- | --- |
| Head | Broad white strip extend in median interorbicular space and along eyes margin. Palps white at tip | Vertex with pale narrow scales extend along the eyes margin. Palps entirely dark | Vertex with pale narrow scales extend along the eye margin. Palps entirely dark |
| Thorax/scutum | An only longitudinal median white stripe | Three longitudinal stripes and two paired shorter brownish | Three longitudinal stripes and two paired shorter brownish |
| Subspiracular area | Patch of white scales present | Patch of pale scales present | Pale scales absent |
| Fore tarsum | Narrow basal white band on tarsomere 1 and 2 | Narrow basal white band on tarsomere 1, sometimes on tarsomere 2 | Narrow basal white band on tarsomeres 1 and 2 |
| Mid tarsum | Narrow basal white band on tarsomere 1 and 2 | Narrow basal white band on tarsomeres 1-3 | Narrow basal white band on tarsomeres 1 and 2 |
| hind tarsum | Broad basal white band on tarsomeres 1-4. Tarsomere 5 entirely white | Narrow basal white band on tarsomeres 1-5 | Narrow basal white band on tarsomeres 1-3. Tarsomere 5 entirely dark |
| Abdomen | Tergum with a thin basal white stripe | Tergum without a stripe but with a basomedian and basolateral pale area | Tergum without a stripe but with a basomedian and basolateral pale area |
| **Characteristics of larvae** | ***Aedes albopictus*** | ***Aedes koreicus*** | ***Aedes japonicus*** |
| Antenna spicules | Absent | Present | Present |
| Antennal setae 1A | Single | Multiple | Multiple |
| Pecten and teeth | All pecten teeth with multiple denticles evenly spaced ahead hair tuft | All pecten teeth with multiple denticles evenly spaced ahead hair tuft | Detached simple pecten teeth beyond hair tuft |
| Comb scales on VIII segment | Apical spine and weak subapical spines | Pectinate terminally and fringed laterally | Pectinate terminally and fringed laterally |

**Table S3**
