# Supplementary figures and images for: Distribution and habitat characterization of the recently introduced invasive mosquito Aedes koreicus [Hulecoeteomyia koreica], a new potential vector and pest in north-eastern Italy
Source: Parasit Vectors. 2013 Oct 10;6:292. doi: 10.1186/1756-3305-6-292 (PMC3852218; doi:10.1186/1756-3305-6-292)

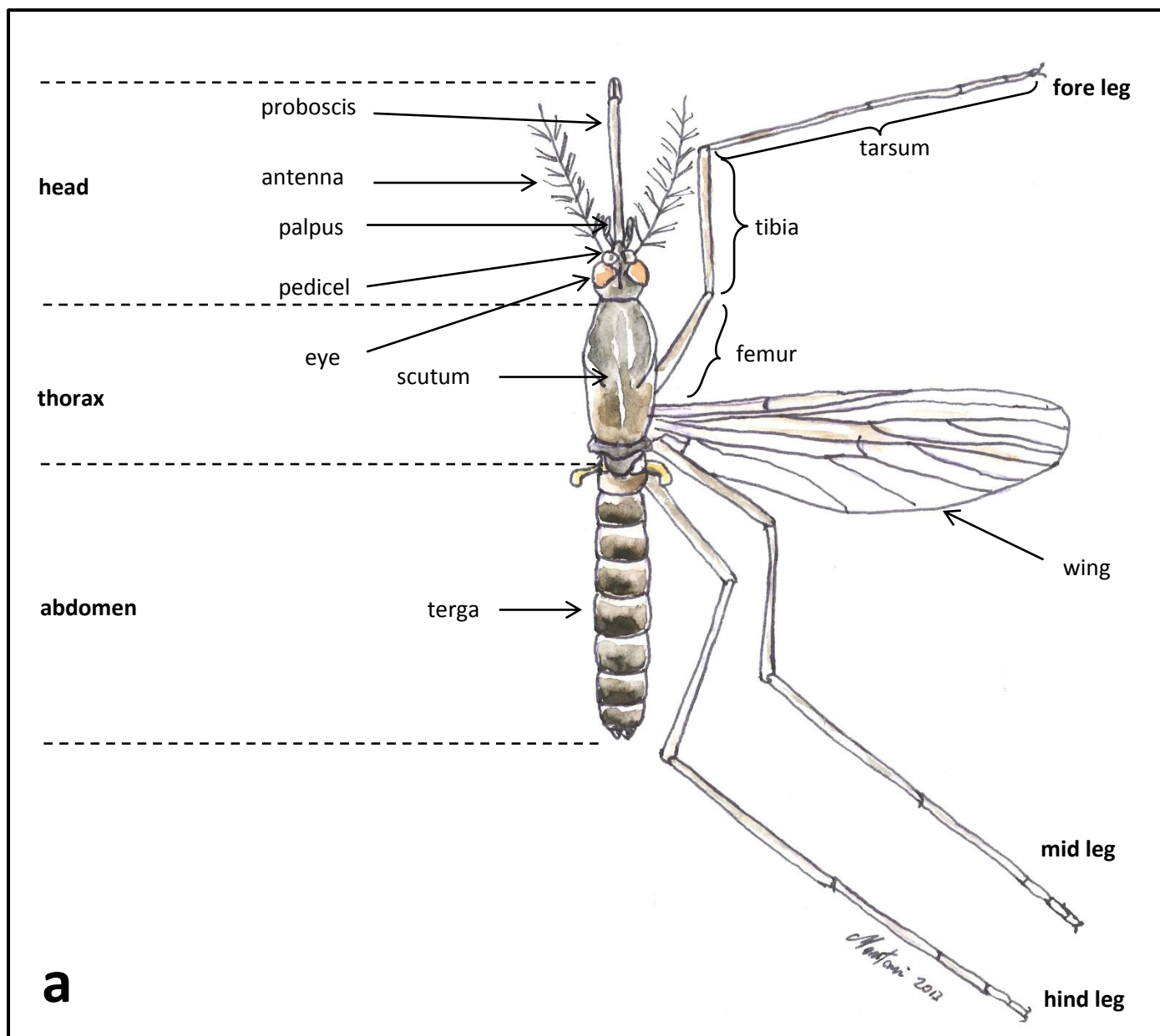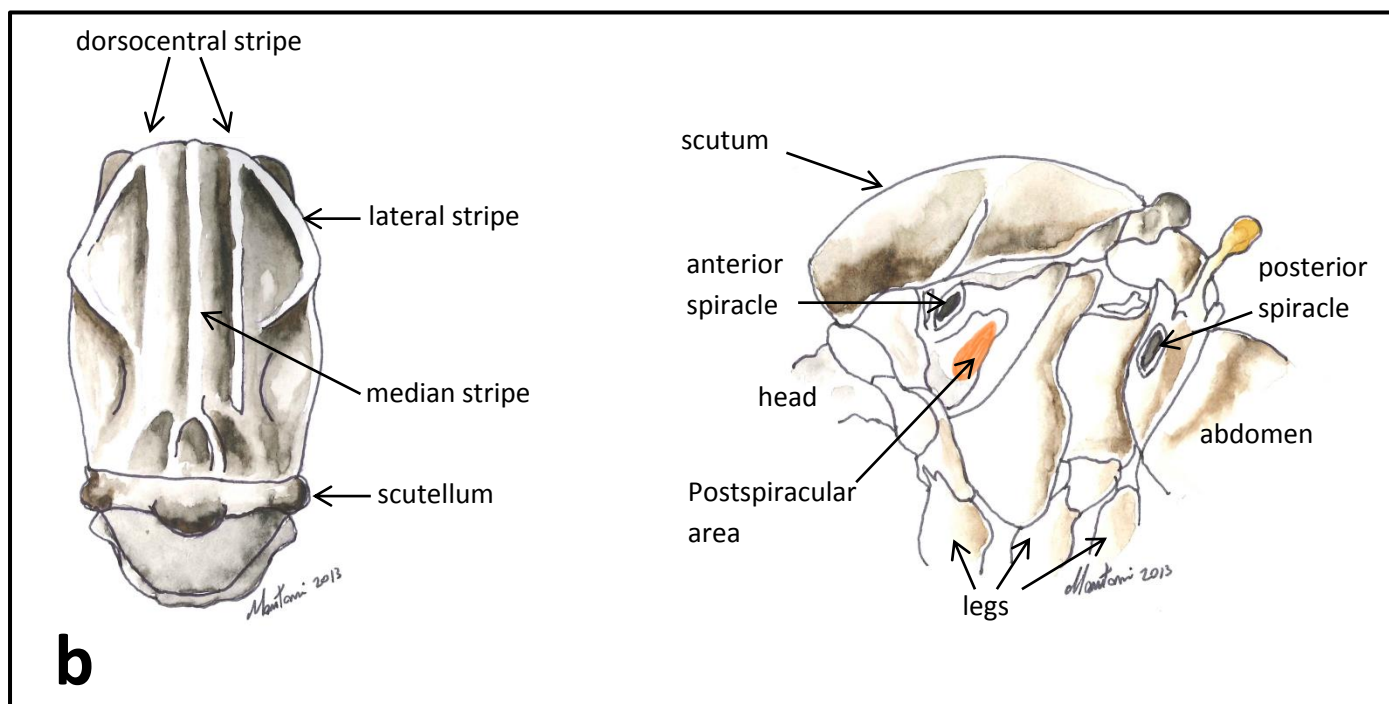

Supplement: Additional file 3 — Drawings of the main characteristics considered for the identification of female culicinae mosquito. a - general aspect of a female mosquito; b – dorsal and lateral view of thorax. The arrows point only the characteristics useful for the identification of Aedes koreicus and Aedes albopictus. [file 1756-3305-6-292-S3.pdf]

**c**

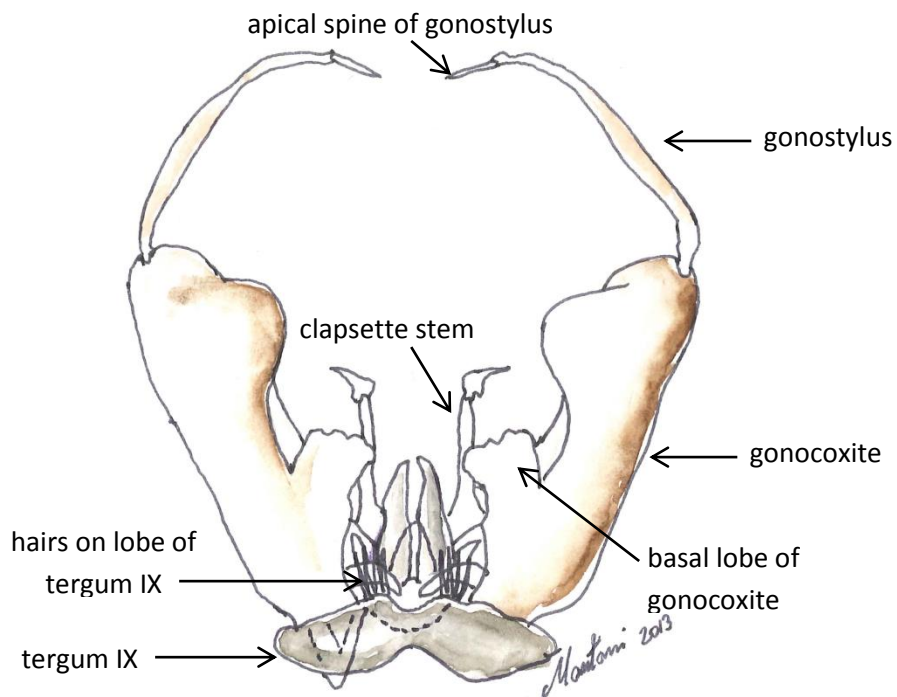

**d**

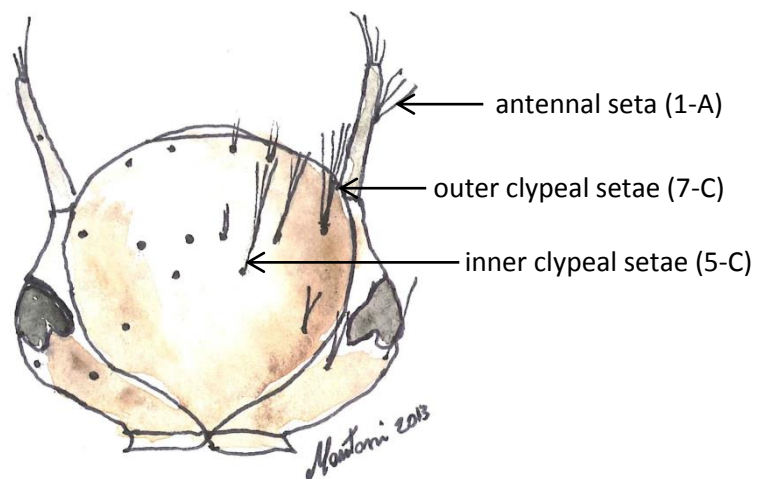

**e**

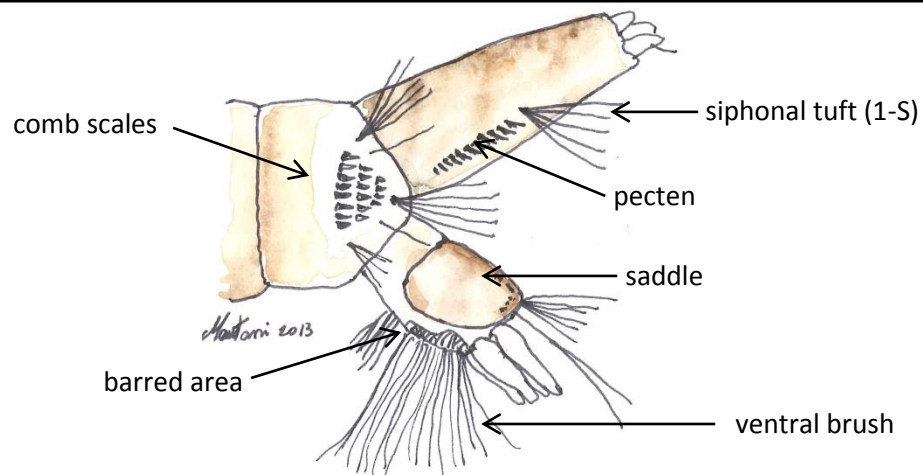

Supplement: Additional file 4 — Drawings of main characteristics considered for the identification of larvae and male of Aedes species. c – hypopygium of Aedes; d – head of culicinae larvae; e – lateral view of the distal part of the larval abdomen of Aedes/Ochlerotatus species. The arrows point only the characteristics useful for the identification of Aedes koreicus and Aedes albopictus. [file 1756-3305-6-292-S4.pdf]

**Figure 5**

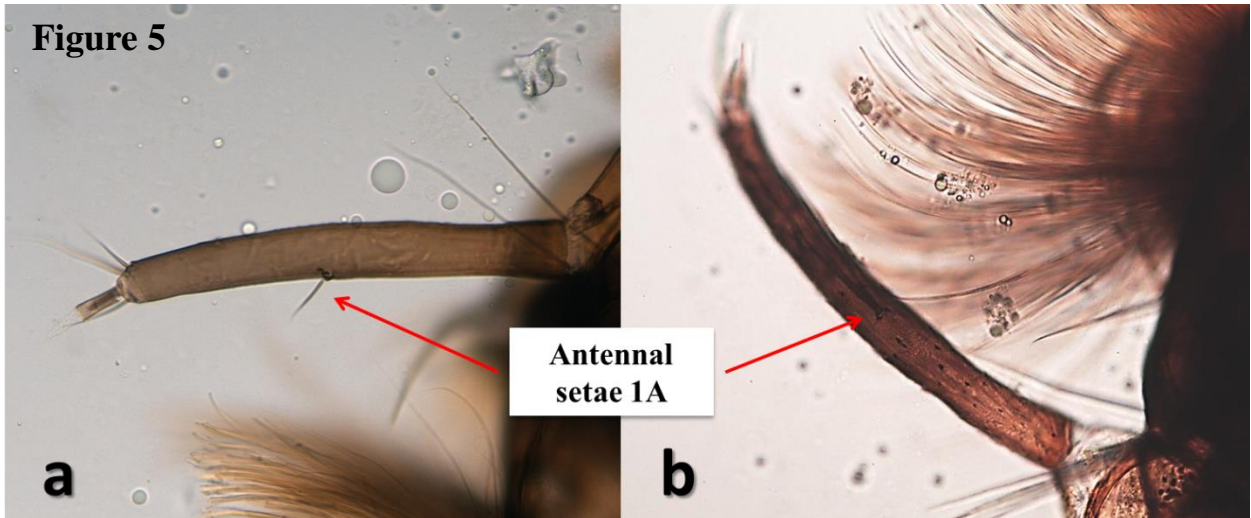

**Figure 6**

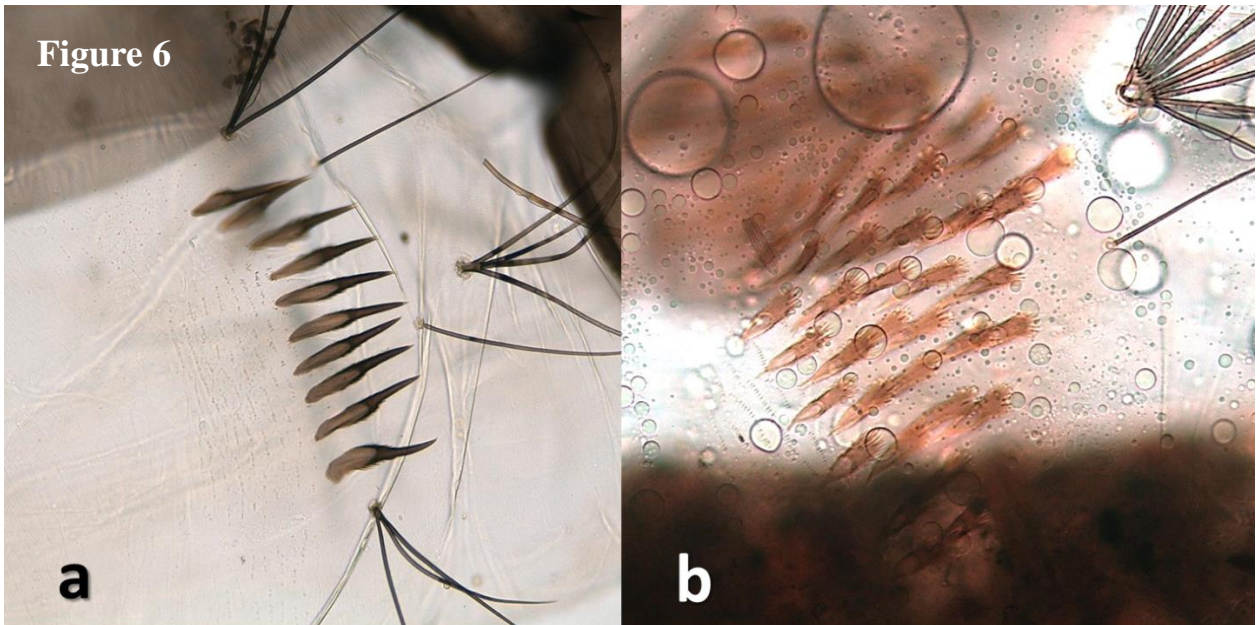

**Figure 7**

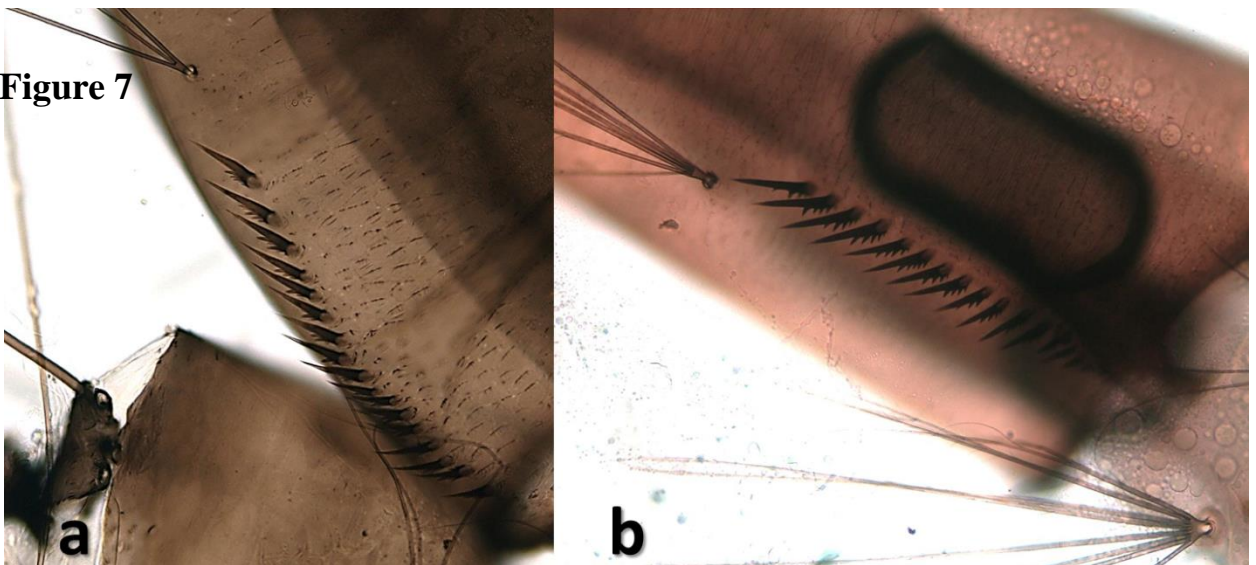

Supplement: Additional file 5 — Comparison between larval features of Aedes albopictus and Aedes koreicus. Difference between Aedes albopictus (a) and Aedes koreicus (b); Figure S5, antennae and antennal setae 1A; Figure S6, comb scales; Figure S7, pecten. [file 1756-3305-6-292-S5.pdf]

**Figure 8**

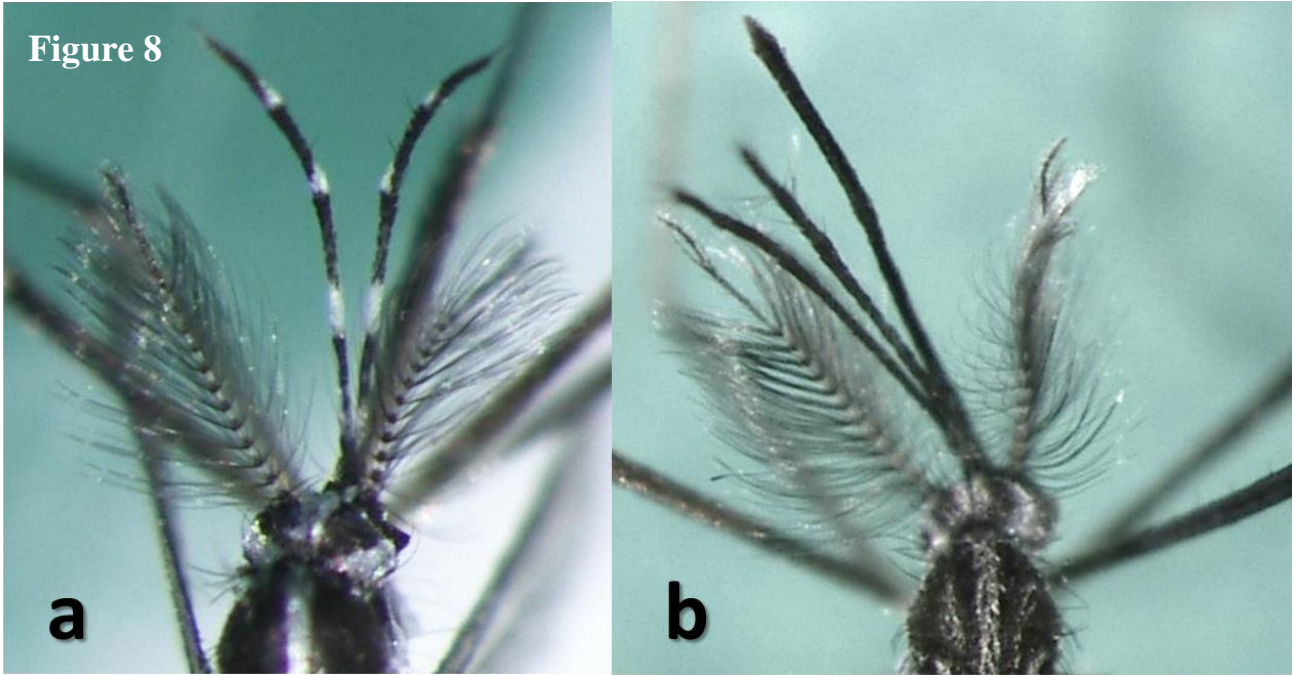

**Figure 9**

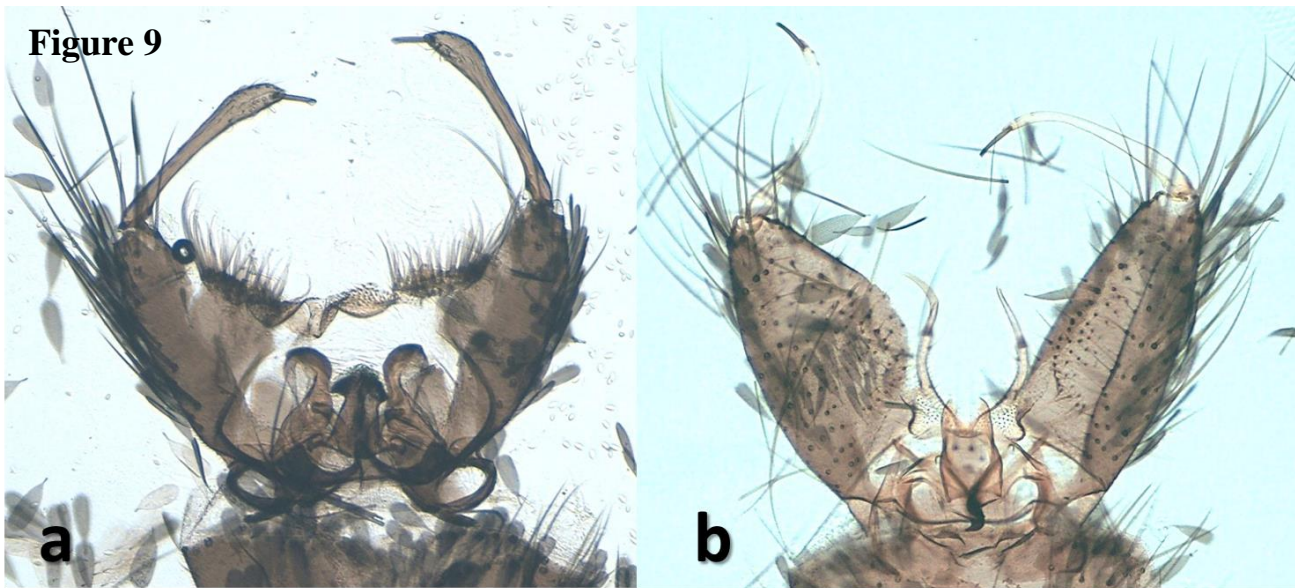

Supplement: Additional file 6 — Comparison between male features of Aedes albopictus and Aedes koreicus. Difference between Aedes albopictus (a) and Aedes koreicus (b); Figure S8, palps and proboscids; Figure S9, hypopygium. [file 1756-3305-6-292-S6.pdf]

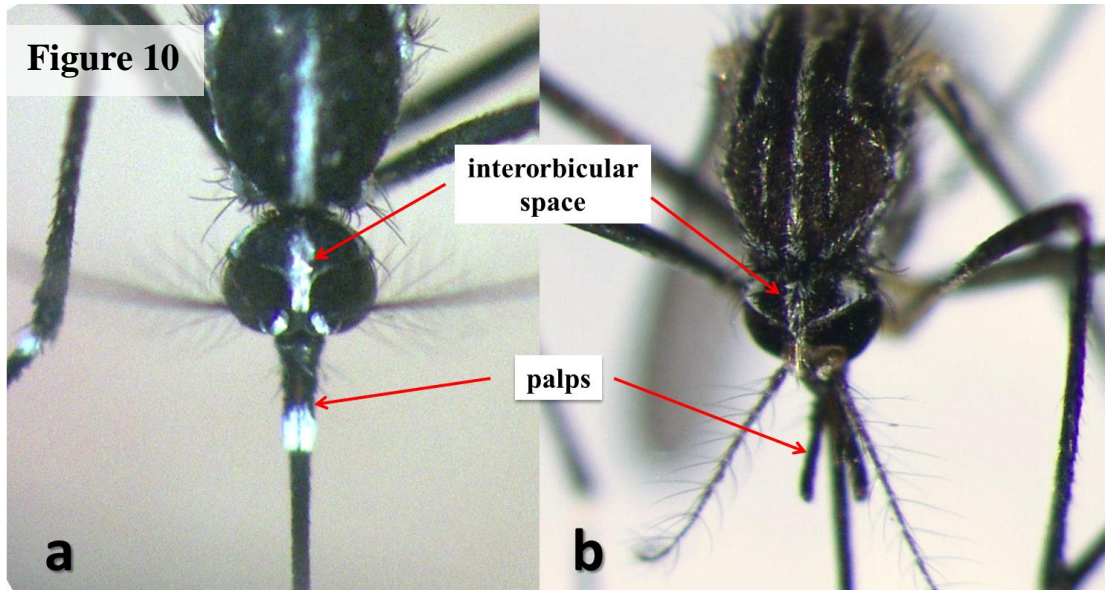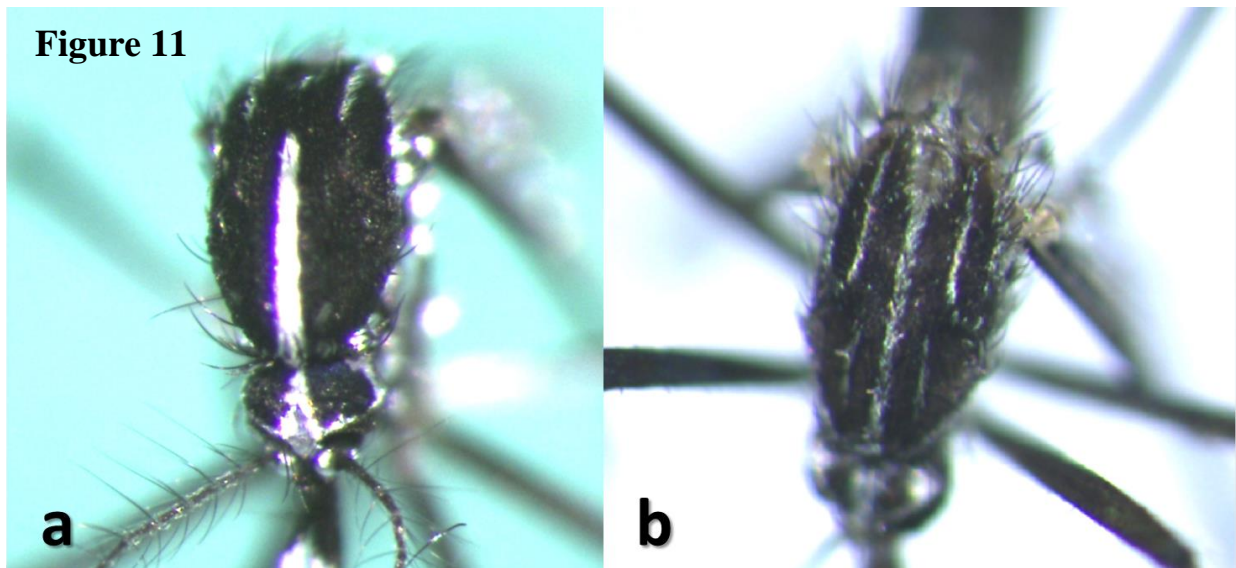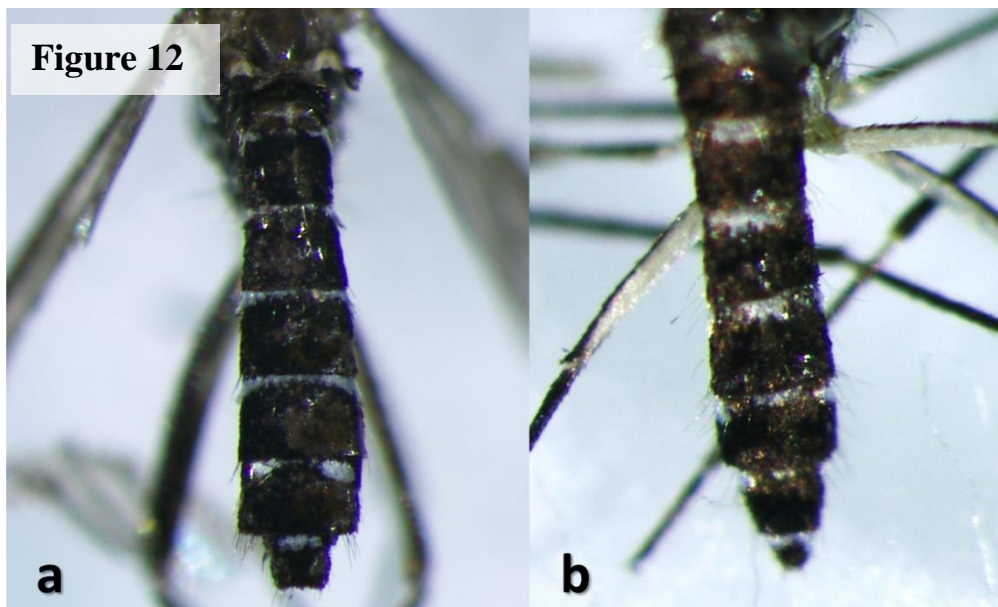

Supplement: Additional file 7 — Comparison between female features of Aedes albopictus and Aedes koreicus. Difference between Aedes albopictus (a) and Aedes koreicus (b); Figure S10, palps and interorbicular space; Figure S11, ornamentation on scutum; Figure S12, abdomen. [file 1756-3305-6-292-S7.pdf]

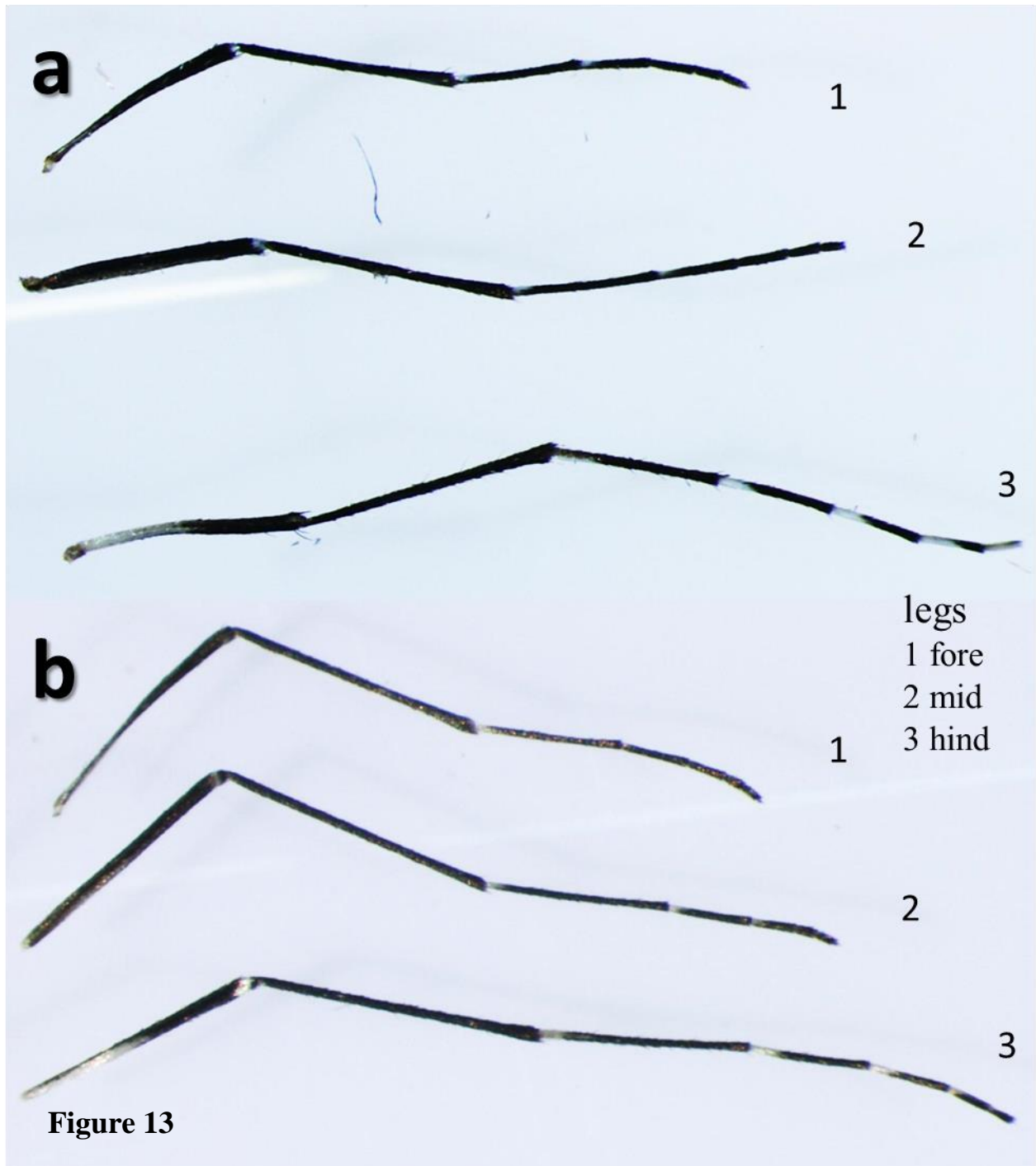

Supplement: Additional file 8 — Comparison between legs of Aedes albopictus and Aedes koreicus. Difference between Aedes albopictus (a) and Aedes koreicus (b); Figure S13, 1-2-3 fore, mid and hind legs respectively. [file 1756-3305-6-292-S8.pdf]
